# Supplementary material for: Automating the amino acid identification in elliptical dichroism spectrometer with Machine Learning
Source: PLoS One. 2025 Jan 17;20(1):e0317130. doi: 10.1371/journal.pone.0317130 (PMC11741379; doi:10.1371/journal.pone.0317130)
Supplement: S1 Fig — (PDF) [file pone.0317130.s001.pdf]

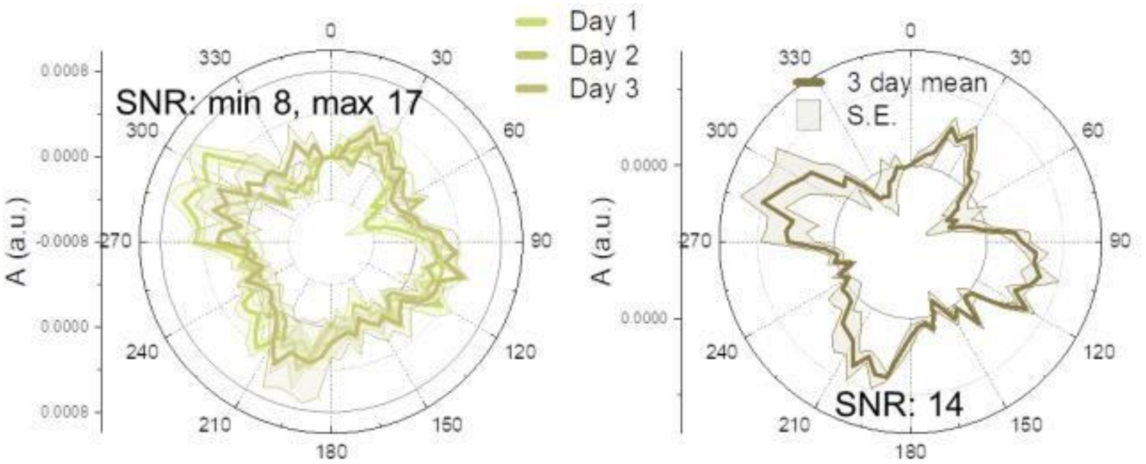

Figure S1: Stability experiment of ED spectrometer over multiple days. This experiment indicates the stability of ED data readout over a 3-day period showcasing low standard error of mean. Signal-to-noise ratio (SNR) is defined as mean/standard deviation.
