# Supplementary material for: Automating the amino acid identification in elliptical dichroism spectrometer with Machine Learning
Source: PLoS One. 2025 Jan 17;20(1):e0317130. doi: 10.1371/journal.pone.0317130 (PMC11741379; doi:10.1371/journal.pone.0317130)
Supplement: S2 Fig — (PDF) [file pone.0317130.s002.pdf]

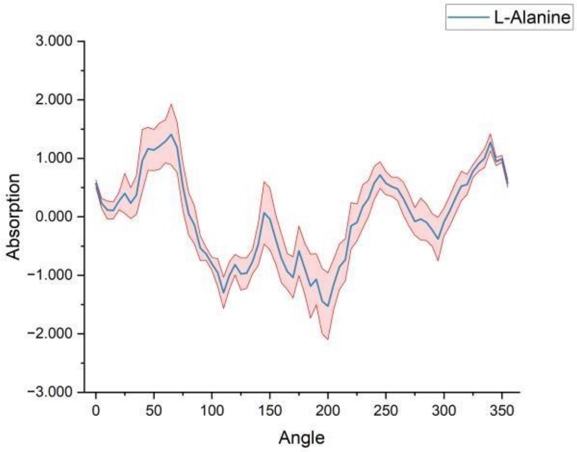
L-Alanine
Absorption
3.000
2.000
1.000
0.000
−1.000
−2.000
−3.000
0 50 100 150 200 250 300 350
Angle

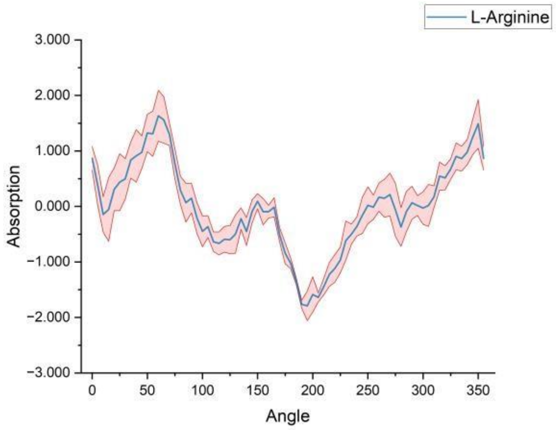
L-Arginine
Absorption
3.000
2.000
1.000
0.000
−1.000
−2.000
−3.000
0 50 100 150 200 250 300 350
Angle

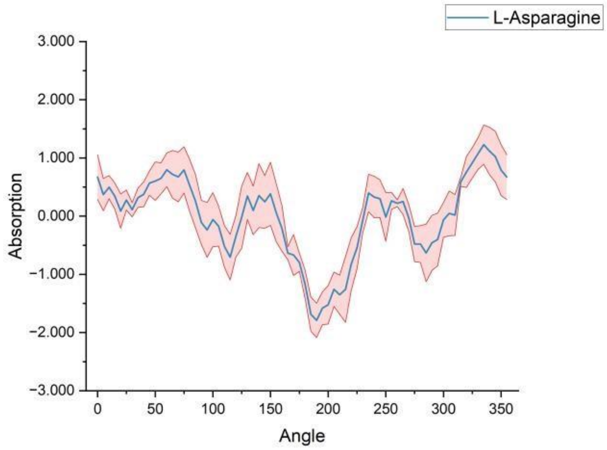
L-Asparagine
Absorption
3.000
2.000
1.000
0.000
−1.000
−2.000
−3.000
0 50 100 150 200 250 300 350
Angle

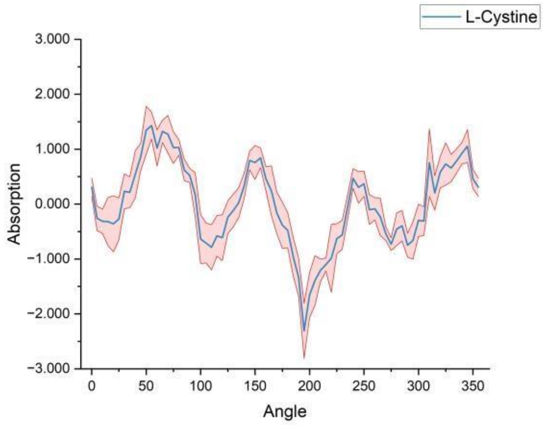
L-Cystine
Absorption
3.000
2.000
1.000
0.000
−1.000
−2.000
−3.000
0 50 100 150 200 250 300 350
Angle

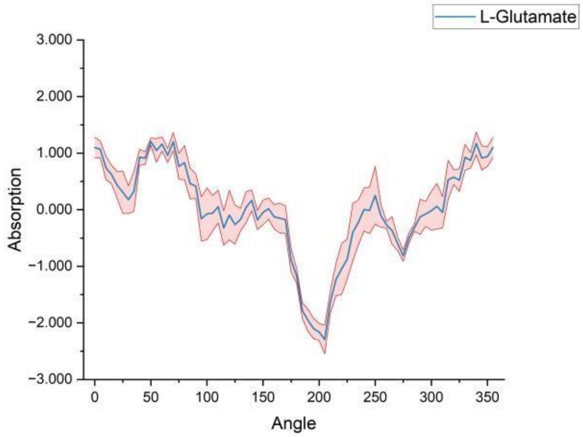
L-Glutamate
Absorption
3.000
2.000
1.000
0.000
−1.000
−2.000
−3.000
0 50 100 150 200 250 300 350
Angle

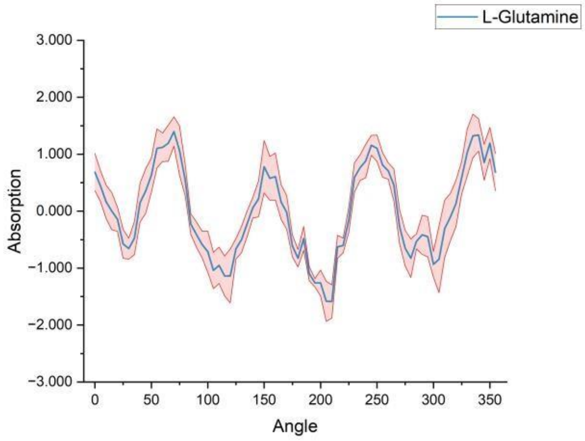
L-Glutamine
Absorption
3.000
2.000
1.000
0.000
−1.000
−2.000
−3.000
0 50 100 150 200 250 300 350
Angle

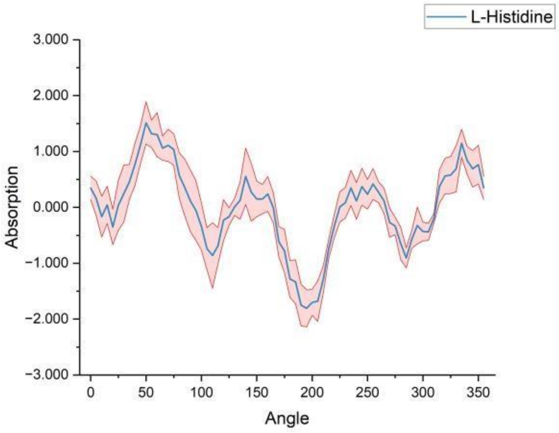
L-Histidine
Absorption
Angle
3.000
2.000
1.000
0.000
−1.000
−2.000
−3.000
0
50
100
150
200
250
300
350

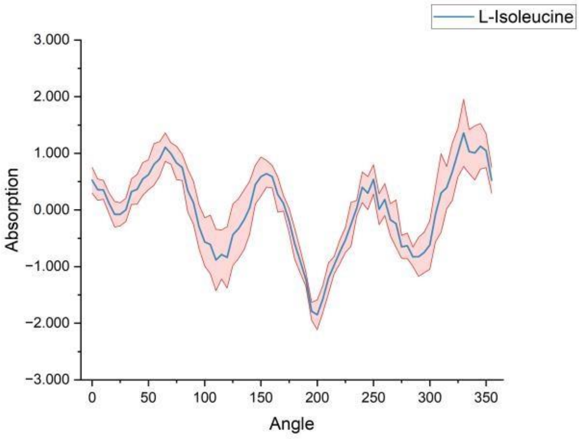
L-Isoleucine
Absorption
Angle
3.000
2.000
1.000
0.000
−1.000
−2.000
−3.000
0
50
100
150
200
250
300
350

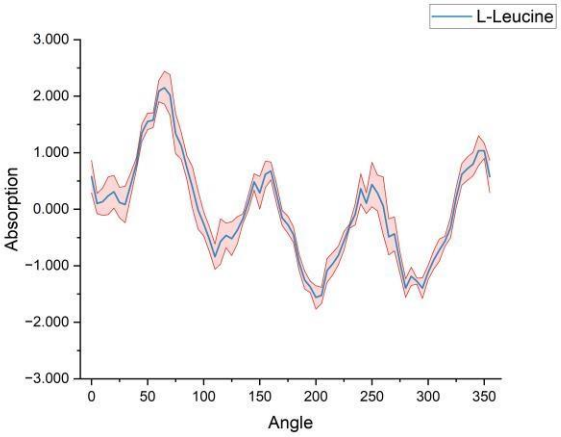
L-Leucine
Absorption
Angle
3.000
2.000
1.000
0.000
−1.000
−2.000
−3.000
0
50
100
150
200
250
300
350

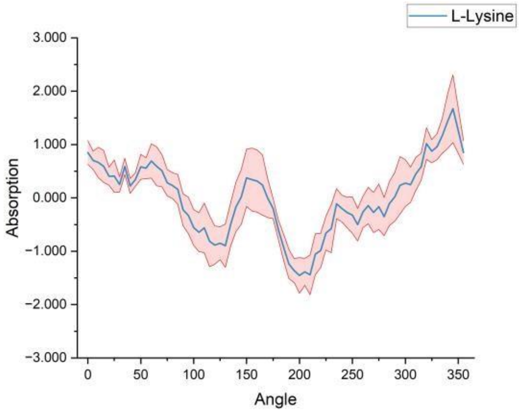
L-Lysine
Absorption
Angle
3.000
2.000
1.000
0.000
−1.000
−2.000
−3.000
0
50
100
150
200
250
300
350

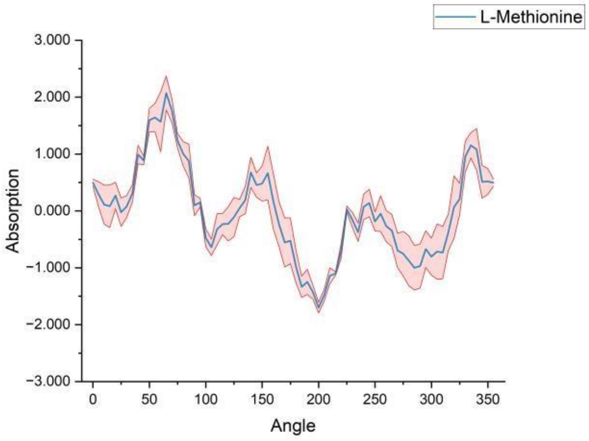
L-Methionine
Absorption
Angle
3.000
2.000
1.000
0.000
−1.000
−2.000
−3.000
0
50
100
150
200
250
300
350

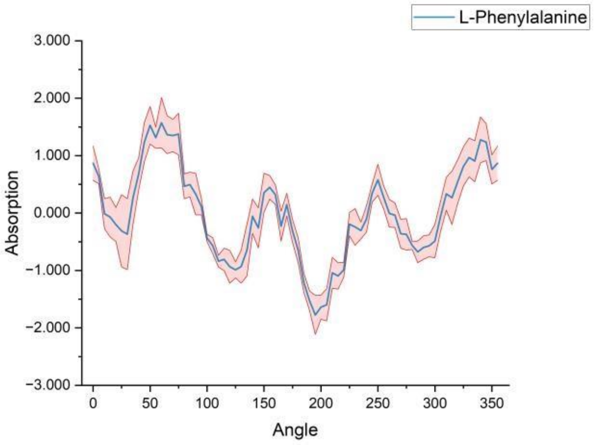
L-Phenylalanine
Absorption
Angle
3.000
2.000
1.000
0.000
−1.000
−2.000
−3.000
0
50
100
150
200
250
300
350

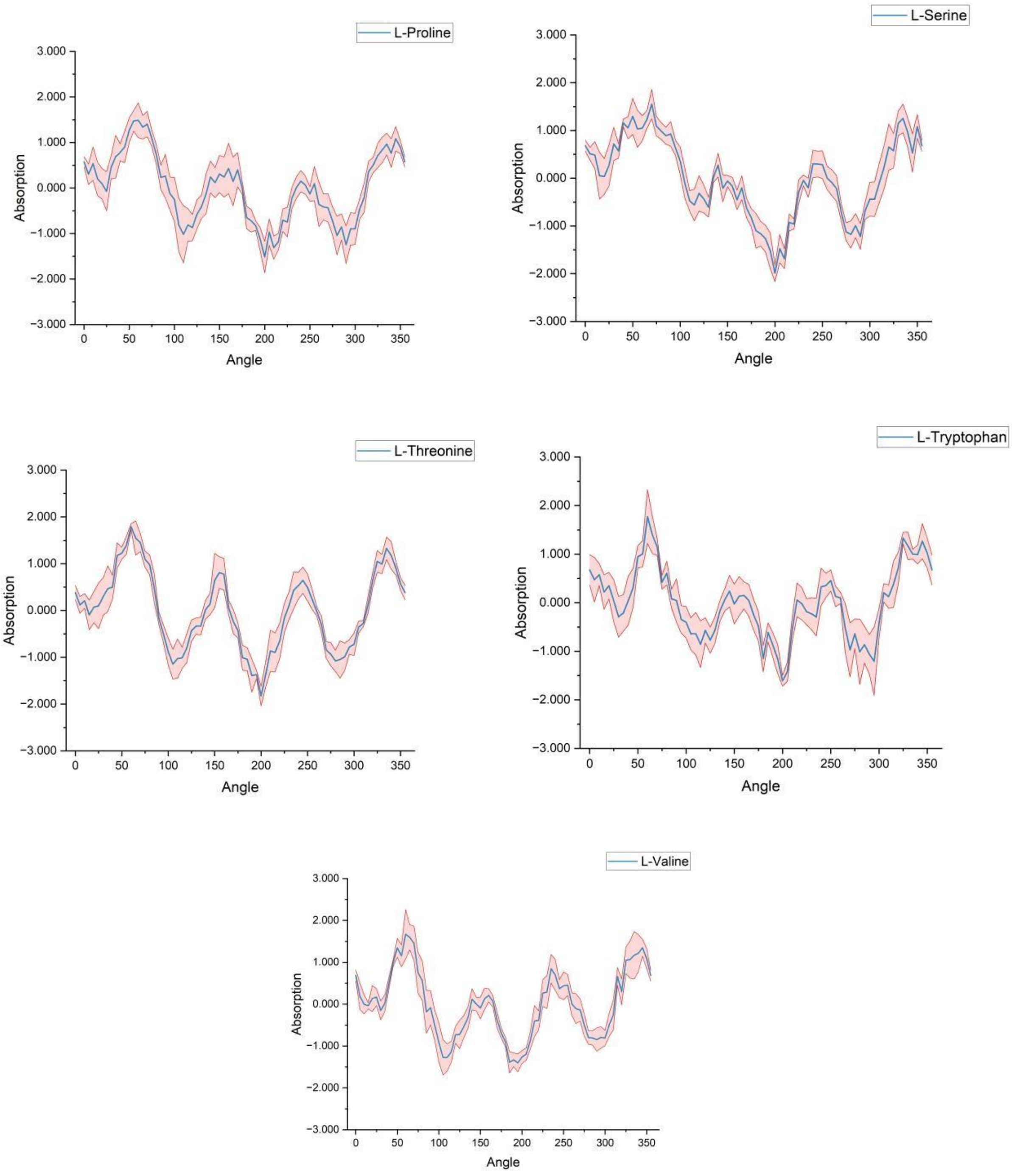

Figure S2: Baseline corrected and normalized absorption curves for all 17 amino acids. Absorption curve for 17 amino acids indicated by solid blue line with standard error of mean shaded in red.
