# Supplementary material for: Automating the amino acid identification in elliptical dichroism spectrometer with Machine Learning
Source: PLoS One. 2025 Jan 17;20(1):e0317130. doi: 10.1371/journal.pone.0317130 (PMC11741379; doi:10.1371/journal.pone.0317130)
Supplement: Attachment — Submitted filename: PONE-D-24-43688_Review report.pdf [file pone.0317130.s003.pdf]

Manuscript title: Automating the Amino Acid Identification in Elliptical Dichroism Spectrometer With Machine Learning

Summary: Amino acid identification is essential across disciplines such as biochemistry, pharmaceutical research, and medical diagnostics. Traditional methods, including mass spectrometry, require extensive sample preparation and are time-consuming, complex, and costly. This study introduces an innovative Machine Learning (ML) approach for amino acid identification based on the unique absorption profiles captured by Elliptical Dichroism (ED) spectrometry. Through advanced data pre-processing and ML pattern recognition techniques, this approach demonstrates the feasibility of distinguishing between amino acids efficiently. Preliminary results indicate promising accuracy, suggesting that ML could significantly enhance amino acid analysis and detection, offering a faster, more accessible alternative to conventional methods.

General comments: The authors have well presented a compelling case for using machine learning in amino acid identification via unique absorption profiles from Elliptical Dichroism (ED) spectrometry. I have few suggestions to strengthen the clarity and impact of this manuscript (MS):

Comment 1: The introduction (page 2-3) concisely highlights the importance of amino acid identification and the limitations of traditional methods, but emphasizing the specific challenges these methods face (e.g., sensitivity, throughput, sample volume) to underscore why a new approach is needed. A table can be added in the MS with a comparison between traditional and new technique on the basis of sensitivity, accuracy and repeatability for all amino acids. It will be helpful to draw the attention of a reader and provide a broad idea about the measurement techniques.

Comment 2: Page 3, Section 2.1 Data Acquisition. Here authors collected 628 absorption profiles of 17 soluble amino acids. But, the data count is unevenly distributed in the table 1. Is there any specific reason for creating this random distribution? What will be the error in the results, if we compare the identification results for L-Arginine and L-Tryptophan with the highest (142) and lowest (12) data count respectively?

Comment 3: Page 5, Figure 2. The curves shown in the figure 2 are qualitatively same, before and after the baseline correction. Here, I am concerned about the fluctuation in the data when changing the angle with a step size of 5°. How authors differentiate between the absorption profiles for 17 amino acids with such a large fluctuation in the data? A figure with normalized and corrected absorption profiles (with error bars) for all amino acids can be added in the MS to visualize the difference between all of them.

Comment 4: Section 3 Results and Discussion: This MS is based on ML and computational developments, so it might be difficult for a biophysicist, biochemist or a biologist to understand it. I request the authors to briefly explain how the absorption profile is used in the study. Whether intensity at each rotation angle is used to differentiate between amino acids or whole profile is compared? Are there any physical equations used to deduce any specific parameter for comparison? This information will provide a physical understanding of the technique and make this MS more reader’s friendly and increase the reach to biophysicist, biochemist and biologists.

Comment 5: This MS is based on L-enantiomers but, what will happen if an unknown sample composed of D or mixture of L and D enantiomers? If authors performed any experiments to quantify it then it will be nice to mention it in the MS. According to their expertise authors may comment on it and mentioned it in the conclusion as future scope of this work.

Comment 6: The work is focused on two amino acids (L-Alanine and L-Arginine) with large data counts. So, generalizing the study as mentioned on page 1, abstract "….this study presents a pioneering Machine Learning (ML) approach…." And page 11, conclusion "…we successfully demonstrate the potential of an ML algorithm to precisely identify amino acids through their…" is misleading. It is difficult to generalize the study with inadequate and limited data for other amino acids. The efficiency of existing model might be different for identifying an amino acid from a large even data set for all type of amino acids.

Comment 7: Authors may briefly mention the future directions, limitations and practical applications where this technique might directly benefit biochemistry, pharmaceuticals, or diagnostics.
